# Factors associated with urinary diversion and fatality of hospitalised acute pyelonephritis patients in France: a national cross-sectional study (FUrTIHF-2)

**DOI:** 10.1017/S0950268823001504

**Published:** 2023-09-18

**Authors:** Leslie Grammatico-Guillon, Emeline Laurent, Joseph Fuhrman, Christophe Gaborit, Maxime Vallée, Aurélien Dinh, Albert Sotto, Franck Bruyere

**Affiliations:** 1 Public Health and Prevention Department, Unit of Regional Clinical Epidemiology, Teaching Hospital of Tours, Medical School, University of Tours, Tours, France; 2 Public Health and Prevention Center, Unit of Regional Clinical Epidemiology, Teaching Hospital of Tours, Research Team “Education, Ethics and Health”, University of Tours, Tours, France; 3 Public Health and Prevention Department, Unit of Regional Clinical Epidemiology, Teaching Hospital of Tours, Tours, France; 4 Service of Urology, Teaching Hospital of Poitiers, Medical School, University of Poitiers, Poitiers, France; 5 Service of Infectious Diseases, AP-HP, Medical School, University of Paris, Paris, France; 6 Service of Infectious Diseases, Teaching Hospital of Nimes, Medical School, University of Nimes, Nimes, France; 7 Service of Urology, Teaching Hospital of Tours, Medical School, University of Tours, Tours, France

**Keywords:** acute pyelonephritis, care pathway, hospital discharge data, urinary diversion, epidemiology

## Abstract

Acute pyelonephritis (AP) epidemiology has been sparsely described. This study aimed to describe the evolution of AP patients hospitalised in France and identify the factors associated with urinary diversion and fatality, in a cross-sectional study over the 2014–2019 period. Adult patients hospitalised for AP were selected by algorithms of ICD-10 codes (PPV 90.1%) and urinary diversion procedure codes (PPV 100%). 527,671 AP patients were included (76.5% female: mean age 66.1, 48.0% *Escherichia coli*), with 5.9% of hospital deaths. In 2019, the AP incidence was 19.2/10,000, slightly increasing over the period (17.3/10,000 in 2014). 69,313 urinary diversions (13.1%) were performed (fatality rate 6.7%), mainly in males, increasing over the period (11.7% to 14.9%). Urolithiasis (OR [95% CI] =33.1 [32.3–34.0]), sepsis (1.73 [1.69–1.77]) and a Charlson index ≥3 (1.32 [1.29–1.35]) were significantly associated with urinary diversion, whereas *E. coli* (0.75 [0.74–0.77]) was less likely associated. The same factors were significantly associated with fatality, plus old age and cancer (2.38 [2.32–2.45]). This nationwide study showed an increase in urolithiasis and identified, for the first time, factors associated with urinary diversion in AP along with death risk factors, which may aid urologists in clinical decision-making.

## Introduction

Acute pyelonephritis (AP) is a bacterial infection of the kidney, which is very frequent in the general population worldwide, requiring hospitalisation of patients in 10–30% of cases [1–5]. Published rates of hospitalisation for urinary tract infections (UTIs), including AP, vary worldwide. In Japan, in 2012, the incidence was 6.8 per 10,000 for men and 12.4 for women but ranged from 2 to 7 per 10,000 according to age and sex in Spain over the 2000–2015 period [1, 6]. Similar variation in the incidence of AP has been recorded in the United States, Canada, and South Korea [5, 7, 8]. In France, AP generated 88,159 visits to emergency departments in 2019 [9], and nearly one-quarter of in-hospital UTIs were due to AP [10]. Despite the related morbidity, few studies have described a care pathway for AP, and the frequency of hospitalisations is poorly documented, especially where a diversion was necessary.

To date, no comprehensive studies have been performed in France to accurately describe the incidence of hospitalised AP, its care pathway, and outcomes, whereas they already have been intensively studied in other Western countries [5, 7]. Cases requiring hospitalisation are the most severe and complicated presentations [2], particularly those with hydronephrosis requiring urinary diversion by introducing a retrograde ureteral stent (ureteral or double J stent) or by percutaneous nephrostomy [11, 12]. The frequency of AP requiring ureteral stents remains unknown. Similarly, there are no epidemiological data on the associated factors of diversion and/or poor prognosis (such as death) for patients with AP hospitalised in France.

The French national hospital discharge database (*Programme de Médicalisation des Systèmes d’Information (*PMSI)) based on the mandatory notification of each hospital stay for all French public or private hospitals represents an extraordinary national data repository. It contains objective information, including socio-demographics, comorbidities, and previous hospital visits or admissions. Although initially designed for billing purposes, with the improvement in data processing, it has proved a powerful tool for epidemiological surveillance [10, 13–17]. This database gives a hospital stay summary based on diagnostic codes from the 10th revision of the International Classification of Diseases (ICD-10) and procedure codes from the French Current Procedure Terminology (French CPT). As each patient is assigned a unique number, individuals can be followed over time, allowing robust epidemiological studies at the national level [10, 17–20].

The main objective of this study was to estimate the incidence of hospitalised AP in France, both with and without urinary diversions, and to highlight the factors associated firstly with diversion and secondly with survival, using national hospital discharge data.

## Methods

### Study design and data source

A national cross-sectional study was carried out from 1 January 2014 to 31 December 2019, using national claim data collected from the French diagnosis-related groups (F-DRGs = PMSI); the study ceased the year before the onset of the COVID-19 pandemic.

### Study population, case definition, and outcomes

The study population included all adult patients (≥ 18 years old) hospitalised for AP in France over the study period, identified by specific ICD-10 codes (Supplementary material S1). AP in children was excluded due to their different clinical presentation and care pathways and outpatient stays of <24 h.

An algorithm to select patients with UTI was previously validated with a predictive positive value (PPV) of 70.4% [10]. A validation step, focusing on a sample of 150 AP cases, was performed by a clinical reviewer with a second physician check if necessary to define the performance parameters of the specific AP algorithm: PPV 90.6%, with a 95% confidence interval (95% CI) ranging from 84.8% to 94.8%.

Urinary diversions were identified on the same sample, according to specific procedure codes (Supplementary material S1), with excellent performance parameters: PPV of 100% [96.9–100], sensitivity (Se) of 90.9% [75.7–98.0], specificity (Sp) of 100% [96.9–100], and predictive negative value (PNV) of 97.5% [92.9–99.5] (Supplementary material S2). The algorithm also displayed good performance parameters when considering obstructive cases in primary diagnosis as the gold standard (Supplementary material S3).

The AP care episode was defined as all hospitalisations related to the AP or the urinary stent procedure (change in stent) occurring within three months after discharge from the index hospitalisation and up to 28 days after discharge from the last stay within this 3-month period.

Socio-demographic characteristics (age and sex) were recorded along with pre-existing comorbidities (Charlson index and detailed comorbidities [21]). Obstructive processes such as urinary stone, microbiology result, sepsis, and intensive care unit stay were also documented, as well as the leading cause of hospitalisation defined by the hospital discharge resume. We assumed that the leading cause of hospitalisation, which could be AP but also another condition, was associated with the leading cause of death occurring during the hospital stay.

### Morbidity–mortality

The yearly in-hospital incidence rates of AP, overall and stratified by age and sex, were calculated as the number of hospitalised patients with AP in one year divided by the French adult population during the same year, as reported by the National Institute of Statistics and Economic Studies *(INSEE).* The overall incidence of AP was estimated by averaging the data for each year. Likewise, the hospital case fatality rate for AP was expressed as the number of deaths that occurred during an AP care episode, divided by the number of hospitalised patients with the condition.

### Statistical analyses

The characteristics of the overall study population were stratified according to the presence of a urinary diversion. Continuous variables were described using the mean ± standard deviation (SD) or the median with the minimum, maximum, 1st, and 3rd quartiles. Qualitative variables were expressed as frequency and per cent. Multivariate analyses were performed using logistic regression models. For each outcome, all variables with p < 0.2 in bivariate analysis were included in the initial model. The final model was obtained using a descending stepwise selection. Statistical significance was defined at the threshold of p < 0.05. The same modelling was performed to identify factors associated with hospital AP fatality, including the primary diagnosis as a cofactor. Statistical analyses were performed using the SAS version for Microsoft Windows (SAS Institute, Cary, NC) and Microsoft Excel, version 2010.

## Results

**Figure 1.**
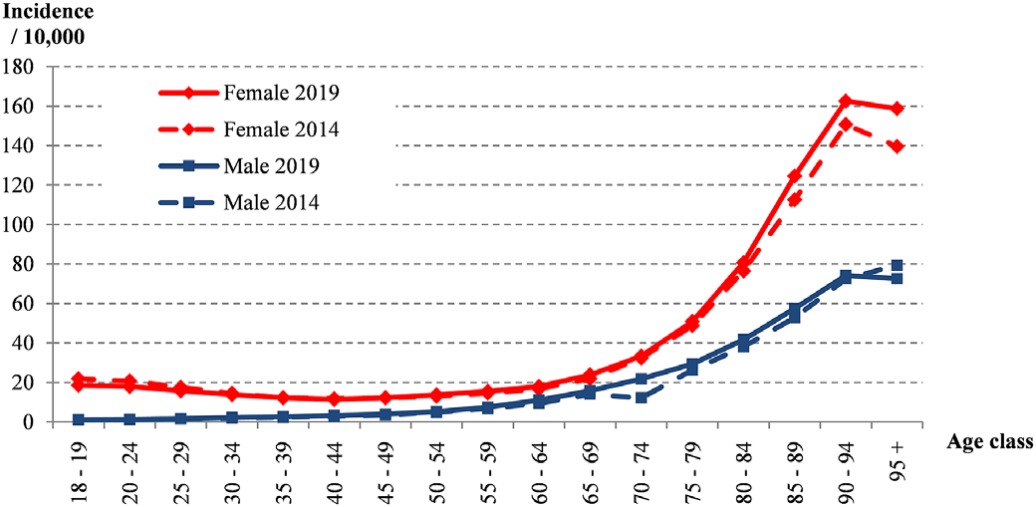
Incidences of hospitalised AP by age and sex, France, 2014 vs. 2019.

**Figure 2.**
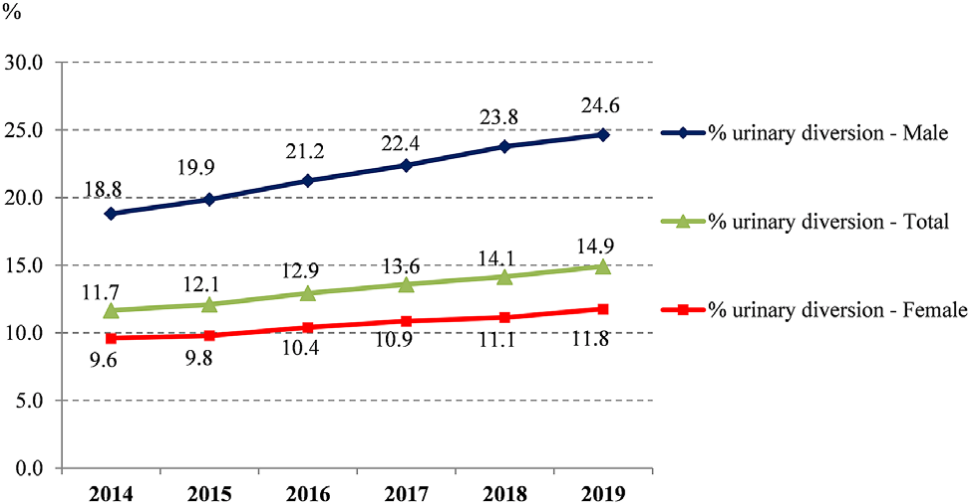
Kidney diversion rate of patients hospitalised with AP, by sex, France, 2014–2019.

**Figure 3.**
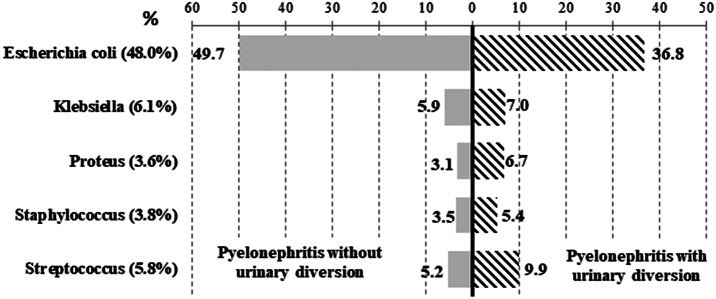
Micro-organisms coded in hospitalised AP, according to the presence of a kidney diversion, France, 2014–2019.

**Figure 4.**
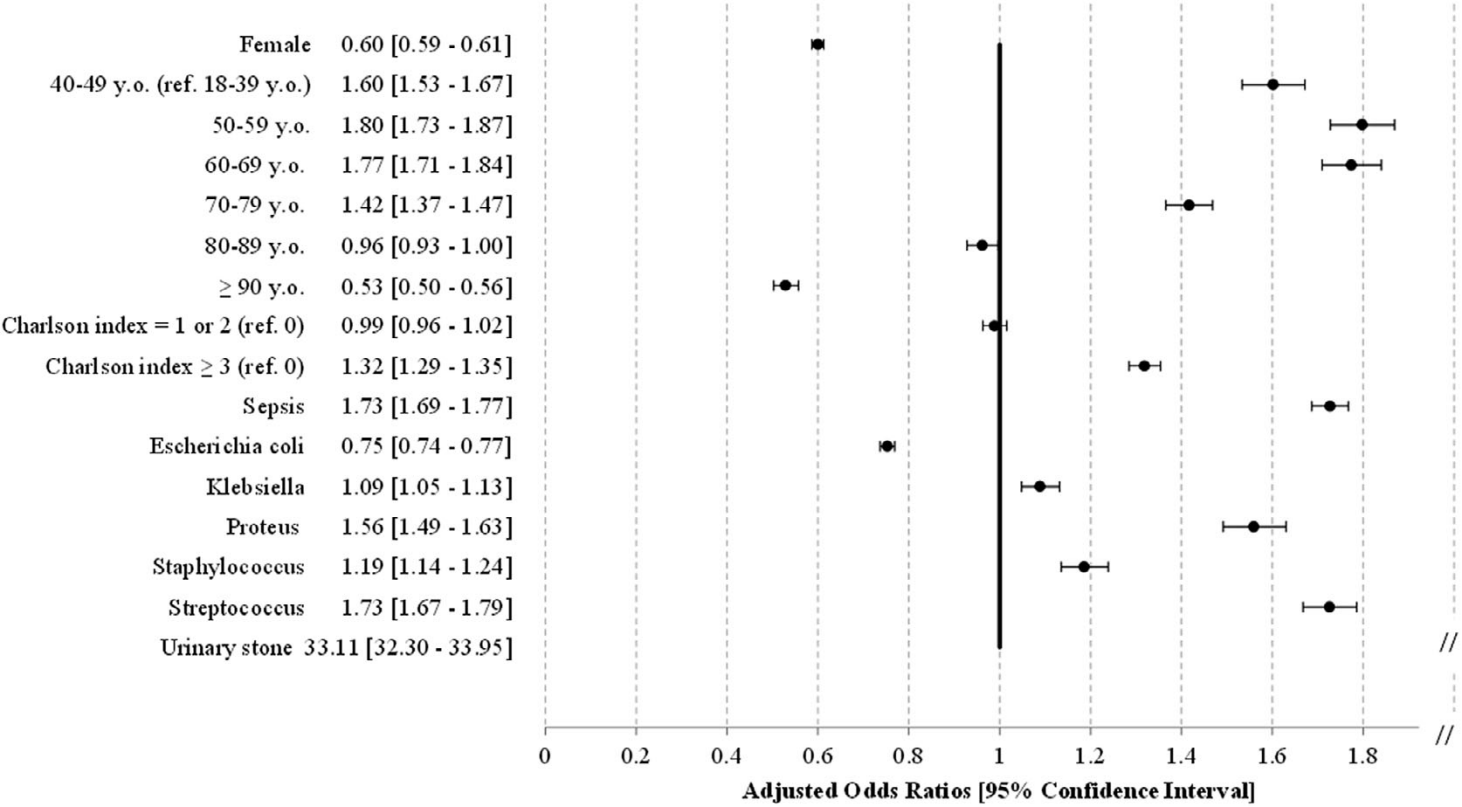
Factors associated with kidney diversion in hospitalised AP, France, 2014–2019.

From 2014 to 2019, 527,671 AP-related hospitalisations were recorded in France and occurred mainly in females (76.5%) or individuals over 60 years old (66.9%) (Figure 1). The mean annual incidence of cases was 18.3/10,000 inhabitants, increasing from 17.3 to 19.2/10,000 during the study period. Urinary diversion accounted for 69,313 patients (13.1%) and increased over the period (Figure 2). This procedure was mainly performed using a double J stent (85.1%), in patients with a urinary stone (48%), and overall, for 74% of the latter group. In total, 198,011 (37.5%) had at least one comorbidity accounting for a Charlson index of 1 or higher, with the main comorbidities being renal disease (15.3%) or cancer (13%) (Table 1). Among those with a urinary diversion, renal disease and cancer accounted for 18.1% and 19.4% of subjects, respectively. The most common microorganism coded in the hospital resume for AP was *Escherichia coli* (48.0% of the overall population study) (Table 1); a similar proportion (36.8%) of those with diversion were positive for this species (Figure 3). Apart from the presence of a urinary stone (OR 33.1 [32.3–34.0]), other factors associated with urinary diversion were as follows: age 50 to 69 years, a Charlson score of ≥3, the presence of sepsis, and isolation of bacteria other than *E. coli* (Figure 4).

**Table 1.** Baseline characteristics of the study population, France, 2014–2019

		Total		Urinary diversion performed	
		*N*	%	*N*	%
Patients		527,671	100	69,313	100
Female		403,475	76.5	42,005	60.6
Age, mean ± std		66.1 ± 21.9		63.6 ± 18.0	
	18–39	87,752	16.6	8,294	12
	40–49	37,639	7.1	6,827	9.8
	50–59	49,148	9.3	10,490	15.1
	60–69	70,909	13.4	14,578	21
	70–79	90,066	17.1	13,757	19.8
	80–90	134,529	25.5	12,621	18.2
	≥90	57,628	10.9	2,746	4.0
Charlson index					
*Detailed comorbidities*					
	Renal disease	80,525	15.3	12,535	18.1
	Cancer	68,595	13	13,425	19.4
	Metastasis	22,977	4.4	5,093	7.3
	Dementia	48,519	9.2	2,833	4.1
	Heart failure	42,196	8	2,923	4.2
	Pulmonary disease	38,164	7.2	3,933	5.7
	Paraplegia	17,423	3.3	2,109	3
	Diabetes with complication	15,173	2.9	1,415	2
	Liver disease	11,651	2.2	1,125	1.6
	Severe liver disease	2,583	0.5	181	0.3
	Connective tissue disease	9,785	1.9	754	1.1
	AIDS	1,677	0.3	251	0.4
*3-category score*					
	0	329,660	62.5	44,067	63.6
	1–2	101,780	19.3	12,277	17.7
	≥3	96,231	18.2	12,969	18.7
Sepsis		105,245	19.9	20,103	29.0
	Severe sepsis	44,694	8.5	11,381	16.4
Intensive care unit		42,592	8.1	10,615	15.3
Microbiology					
	*Escherichia coli*	253,382	48.0	25,536	36.8
	*Klebsiella pneumoniae*	32,047	6.1	4,832	7
	Proteus spp.	19,006	3.6	4,613	6.7
	*Staphylococcus* spp.	19,813	3.8	3,740	5.4
	*Streptococcus* spp.	30,688	5.8	6,889	9.9
	No pathogen code retrieved	205,806	39	30,262	43.7
Urinary stone		45,242	8.6	33,449	48.3
Death		31,227	5.9	4,637	6.7

**Figure 5.**
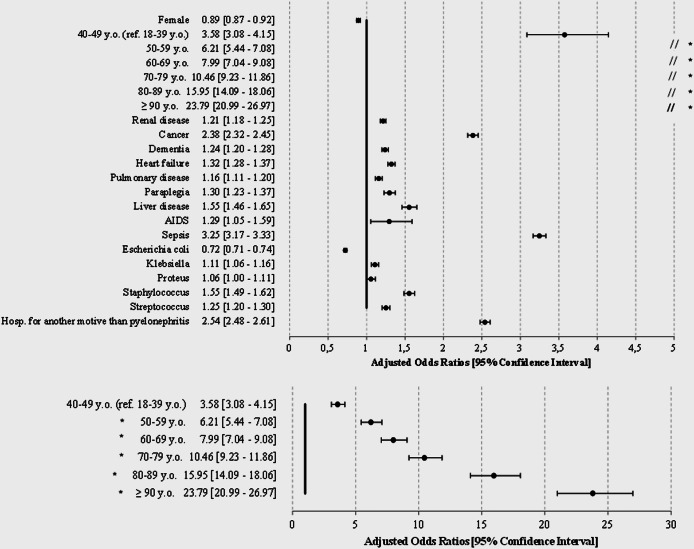
Factors associated with death of patients with hospitalised AP, France, 2014–2019.

Among the 527,671 patients, 31,227 deaths occurred (5.9%), and 6.7% for those with a urinary diversion. The hospital-case fatality was associated with increasing age (Figure 5) from OR = 3.6 [3.1–4.2] between 40 and 49 years to OR = 24.8 [21.0–27.0] above 90, and associated with comorbidities, especially cancer (2.4 [2.3–2.5]) and sepsis (3.3 [3.2–3.3]), independently of the hospitalisation main cause.

## Discussion

This large population-based study allowed studying the burden and predictors of the AP course, including more than 500,000 cases over a long period at a national scale. This study is, to date, one of the only estimations of the incidence of AP-related hospitalisations at this scale. A robust computerised tool using a hospital discharge database algorithm previously validated for UTIs [10] has been applied for AP and urinary diversion, after confirming its performance. In a real-life setting, the incidence estimate of AP-related hospitalisations was 19/10,000 cases, along with diversion recourse and other risk factors resulted in a case fatality of 5.6%.

The incidence of in-hospital AP increased slightly over the 5-year period, which could be related to an ageing population. However, incidence varied substantially by sex and age, likely reflecting specific risk factors for UTIs as suggested by our and other studies [2, 5, 7, 10], in that elderly males, sepsis, and comorbid conditions were primarily associated with AP hospitalisation [2, 11]. Some studies have also reported a proportion of patients hospitalised to be lower than 20% among young people, especially females, and higher in the elderly [2, 10, 22]. Differences in incidence rates varied compared with some other Western countries, where in-hospital AP varied widely and was usually less frequent than reported in France [2, 5, 7, 10, 23]. However, an Asian study found markedly higher hospital rates of 30/10,000 inhabitants [8]. Further studies are necessary to address whether the organisation of healthcare systems and reimbursement rates impact hospitalisation care and outcomes, which could be a significant factor in fostering hospital admission in France.

AP may readily progress to severe urosepsis; renal dilatation requires urgent surgical management [2, 7, 9, 24]. The risk factors for renal dilatation are not ascertained and accepted but represent key information for in-hospital AP that can be used to identify patients requiring urinary diversion and/or at risk of severe presentation [22, 24]. Indeed, for economical and organisational reasons, renal imaging, which could quickly detect dilatation, is not mandatory for all patients with AP. In our study, the urinary diversion rate was estimated at 13%. Further definition of risk factors for renal derivation could aid in the identification of patients who require ultrasound or CT scans in an emergency setting. This was the main aim of the study. The presence of a urinary stone (8.6% of AP, OR > 33) was the first predictor, but one-quarter of such patients did not receive a urinary diversion. Checking for the presence of a urinary stone requires an imaging procedure whose result reports, unfortunately, are not available in the medico-administrative database; thus, we were not able to confirm from their coding the presence of dilatation among these patients with urinary stone without diversion. Moreover, we estimated that the diversion frequency increased over the period and was probably linked to the increasing number of AP with lithiasis. Indeed, the incidence of urinary stones increased in the wider population over the period, from 20 to 22/10,000 individuals. Other patient characteristics such as sex, middle age, a Charlson score of 3 or more, and sepsis were also associated with more urinary diversion in our study. A study from 2007 reported that of 2,408 cases of AP, 87% were women and those aged 15–54 years had a higher ratio of outpatient care [5]. This suggests, similar to our findings, that clinical presentations in younger females result in fewer complications and urinary diversions. The presence of *E. coli* was significantly associated with less urinary diversion. A French commentary by Bruyere et al. found that the presence of *E. coli* did not often result in urinary stones [24], which could explain the less frequent performance of urinary diversion, as this pathogen is frequent in the general healthy population. Concerning the elderly, where diversion is also less performed, the hypothesis could be that pre-existing medical conditions make this group more challenging for surgery, due to more cancer in advanced stages. Moreover, as older subjects could possibly present more frequently with stones but potentially without an associated obstruction, they would not require urinary diversion. These findings merit further investigation.

This study also documented the AP hospital course and revealed factors associated with a higher fatality, such as older age and cancer. To our knowledge, this is the first study to estimate the morbimortality burden of hospitalised AP, especially in terms of case fatality, and further analyses are clearly warranted. This is evident from our estimates of the impact of the medical conditions responsible for hospital stay and contributing to the mortality rate. Indeed, the risk of death markedly increased (aOR 2.6) when the primary diagnosis for hospital stay was not AP, which was subsequently identified as a contributory diagnosis but was not the leading driver for hospital admission.

Several limitations of our study should be mentioned. First, information bias classifying AP versus cystitis could exist, especially for *E. coli*, due to the difficulty for some practitioners to distinguish asymptomatic bacteriuria from a UTI [10, 18, 22, 25]. Nevertheless, the microorganism distribution was similar to the literature with *E. coli* in first rank, followed by other Gram-negative bacilli [10, 26]. However, the species documentation was available for only 60% of the AP cases, which was probably due to a lack of pathogen coding as compared with clinical studies [10, 24]. Community and hospital-acquired infections cannot be distinguished through medico-administrative databases, which also do not allow to study resistance of isolates. This lack of coding, due to a lack of financial incentive, does not allow their reliable use for epidemiological surveillance, as presented by comparative studies [17, 27, 28]. Moreover, hospital stays are sometimes coded by various healthcare practitioners, leading to variation in the coded information [18, 20]. However, validation of the algorithm showed excellent PPV (91%) for AP, which was higher than our UTI algorithm [10], and excellent PPV and sensitivity for urinary diversion (100% and 91%, respectively). Indeed, these situations rely on each physician’s assessment, leading to wide heterogeneity according to the healthcare practitioner and their specialty: a surgeon will likely not have the same judgement as an infectious disease specialist or an intensivist [18, 25, 29]. Eventually, regarding the relatively high fatality rate (6%), the claim data design of the study did not tag the cause of death, although the patient could have had an AP but died primarily from a cancer or diabetes mellitus complication during the stay. We tried to address this limitation by attempting to establish the leading cause of hospitalisation.

This study has several strengths linked to the use of medico-administrative databases, providing a large well-defined real-life population. The reuse of data for epidemiological purposes has been intensively described and demonstrated its reliability, accuracy, and low cost with time-saving, for surveillance when well-designed validated algorithms are utilised [5, 10, 18, 19].

## Conclusion

To our knowledge, this is the first population-based study to describe trends in the incidence of AP, its distribution patterns, and factors associated with the derivation of inpatients with acute pyelonephritis, along with the risk factors for death. With the use of a validated algorithm, this national study based on a real-life large database showed an increase from 2014 to 2019 in urinary diversion for AP management, along with an increasing number of patients with urolithiasis. The factors associated with urinary diversion for AP management were identified as sepsis, being elderly, and having comorbidities, which were also associated with fatality. These factors may be of assistance to urologists for rapid decision-making on the presentation of AP.

## Supporting information

Grammatico-Guillon et al. supplementary material 1 Grammatico-Guillon et al. supplementary material

Grammatico-Guillon et al. supplementary material 2 Grammatico-Guillon et al. supplementary material

Grammatico-Guillon et al. supplementary material 3 Grammatico-Guillon et al. supplementary material

## Data Availability

The data were obtained from the National Research Database of the National Health Insurance Hub, PMSI ATIH. Our study involved the reuse of previously recorded and anonymised data. As a continuation of FUrTIHF-1 [10], this study is part of the protocol that was filed as the FUrTIHF study, which fell within the scope of the French Reference Methodology MR-005 (declarations 2205437v0, 22 August 2018, and 2222651v0, 9 June 2021, endorsed by the Tours Teaching Hospital), which requires neither information nor consent of the included individuals. The FUrTIHF study was registered with the French Data Protection Board (CNIL MR-005 number #2018160620). These data required capacitation from the national level and restrictions concerning the sharing for legal reasons. It may not be shared at the public access. To be able to have access to the data set, you must be authorised and request legal access to the project data set.

## Supplementary material

The supplementary material for this article can be found at https://doi.org/10.1017/S0950268823001504.

## Key findings

The first population-based study of hospitalised AP describes trends in incidence and patterns.

Based on real-life data, we showed an increasing number of hospitalised AP and urinary diversions.

Factors associated with urinary diversion and fatality were sepsis, elderly, and comorbidities.

These identified factors could help the urologist for rapid decision-making.
