# Supplementary material for: Factors associated with urinary diversion and fatality of hospitalised acute pyelonephritis patients in France: a national cross-sectional study (FUrTIHF-2)
Source: Epidemiol Infect. 2023 Sep 18;151:e161. doi: 10.1017/S0950268823001504 (PMC10600899; doi:10.1017/S0950268823001504)
Supplement: Grammatico-Guillon et al. supplementary material 1 — Grammatico-Guillon et al. supplementary material [file S0950268823001504sup001.docx]

**Supplementary material A**

**Case definition of acute pyelonephritis via hospital discharge databases**

(*Programme de Médicalisation des Systèmes d’Information* PMSI)

1. **Acute pyelonephritis (AP) – *PMSI* case definition**

| **At least one ICD-10 code among:** | | |
| --- | --- | --- |
|  | N10 | Acute tubulo-interstitial nephritis |
|  | N11.0 | Nonobstructive reflux-associated chronic pyelonephritis |
|  | N11.1 | Chronic obstructive pyelonephritis |
|  | N12 | Tubulo-interstitial nephritis, not specified as acute or chronic |
|  | N13.6 | Pyonephrosis |

1. **AP with urinary diversion – *PMSI* case definition**

| **At least one procedure code among: (French current procedural terminology CCAM: in French)** | | |
| --- | --- | --- |
|  | JACH001 | Néphrostomie, par voie transcutanée avec guidage échographique et/ou radiologique. |
|  | JACH002 | Néphrostomie par voie transcutanée avec drainage internalisé de la voie excrétrice urinaire, avec guidage échographique et/ou radiologique. |
|  | JACH003 | Néphrostomie, par voie transcutanée avec guidage scanographique. |
|  | JACA002 | Néphrostomie cutanée, par abord direct. |
|  | JBCA001 | Pyélostomie cutanée, par abord direct. |
|  | JCLE001 | Pose d'une sonde urétérale à visée thérapeutique, par endoscopie rétrograde. |
|  | JCLE002 | Pose d'une endoprothèse urétérale, par endoscopie rétrograde. |
|  | JCLH001 | Pose d'une endoprothèse urétérale, par voie transcutanée avec guidage échographique et/ou radiologique. |
|  | JCLD00 | Pose d'une endoprothèse urétérale, par une néphrostomie déjà en place. |

1. **Obstructive process : urinary stone – *PMSI* case definition**

| **At least one ICD-10 code among:** | | |
| --- | --- | --- |
|  | N20 | Calculus of kidney and ureter |
|  | N20.0 | Calculus of kidney |
|  | N20.1 | Calculus of ureter |
|  | N20.2 | Calculus of kidney with calculus of ureter |
|  | N20.9 | Urinary calculus, unspecified |
|  | N13.2 | Hydronephrosis with renal and ureteral calculous obstruction |
| **OR** | | |
| **At least one procedure code among: (French current procedural terminology CCAM: in French)** | | |
|  | JCGE001 | Ablation et/ou fragmentation de calcul de l'uretère pelvien, par urétéroscopie rétrograde |
|  | JCGE002 | Ablation et/ou fragmentation de calcul de l'uretère, par urétéroscopie antérograde par une néphrostomie déjà en place |
|  | JCGE005 | Ablation et/ou fragmentation de calcul de l'uretère iliaque, par urétéroscopie rétrograde |
|  | JCGE006 | Ablation et/ou fragmentation de calcul de l'uretère lombal, par urétéroscopie rétrograde |
|  | JCGA002 | Ablation de calcul de l'uretère lombal, par abord direct |
|  | JCGA003 | Ablation de calcul de l'uretère iliaque, par abord direct |
|  | JCGH004 | Ablation et/ou fragmentation de calcul de l'uretère, par urétrocystoscopie avec guidage échographique et/ou radiologique |
|  | JANE002 | Fragmentation intrarénale de calcul caliciel inférieur avec ondes de choc ou laser [Lithotritie intrarénale], par urétéronéphroscopie |
|  | JANE005 | Fragmentation intrarénale de calcul avec ondes de choc ou laser [Lithotritie intrarénale], par urétéronéphroscopie |
|  | JAGF001 | Ablation de calculs multiples du rein, par voie transcutanée |
|  | JCGC001 | Ablation de calcul de l'uretère pelvien, par coelioscopie ou par rétropéritonéoscopie |
|  | JCGC002 | Ablation de calcul de l'uretère iliaque, par coelioscopie ou par rétropéritonéoscopie |
|  | JCGC003 | Ablation de calcul de l'uretère lombal, par coelioscopie ou par rétropéritonéoscopie |
|  | JCGA001 | Ablation de calcul de l'uretère pelvien, par abord direct |
|  | JCGH001 | Ablation et/ou fragmentation de calcul de l'uretère, par voie transcutanée avec guidage échographique et/ou radiologique |
|  | JCGH005 | Ablation et/ou fragmentation de calcul de l'uretère, par une néphrostomie déjà en place avec guidage échographique et/ou radiologique |
|  | JCEE001 | Refoulement de calcul de l'uretère par sonde urétérale, par endoscopie rétrograde |
|  | JCGG001 | Ablation et/ou fragmentation de calcul de l'uretère, par urétéroscopie antérograde par voie transcutanée |
|  | JCNM002 | Lithotritie extracorporelle de l'uretère, avec guidage radiologique |
|  | JANM001 | Lithotritie extracorporelle du rein, avec guidage radiologique |
|  | JCNM001 | Lithotritie extracorporelle de l'uretère, avec guidage échographique |

1. **Other obstructive process – *PMSI* case definition**

| **At least one procedure code among: (French current procedural terminology CCAM: in French)** | | | |
| --- | --- | --- | --- |
|  |  | JACH001 | Néphrostomie, par voie transcutanée avec guidage échographique et/ou radiologique. |
|  |  | JACH002 | Néphrostomie par voie transcutanée avec drainage internalisé de la voie excrétrice urinaire, avec guidage échographique et/ou radiologique. |
|  |  | JACH003 | Néphrostomie, par voie transcutanée avec guidage scanographique. |
|  |  | JACA002 | Néphrostomie cutanée, par abord direct. |
|  |  | JBCA001 | Pyélostomie cutanée, par abord direct. |
|  |  | JCLE001 | Pose d'une sonde urétérale à visée thérapeutique, par endoscopie rétrograde. |
|  |  | JCLE002 | Pose d'une endoprothèse urétérale, par endoscopie rétrograde. |
|  |  | JCLH001 | Pose d'une endoprothèse urétérale, par voie transcutanée avec guidage échographique et/ou radiologique. |
|  |  | JCLD00 | Pose d'une endoprothèse urétérale, par une néphrostomie déjà en place. |
|  |  | | |
|  | **AND** | | |
|  | **At least one ICD-10 code among:** | | |
|  |  | C52 | Malignant neoplasm of vagina |
|  |  | C53 | Malignant neoplasm of cervix uteri |
|  |  | C54 | Malignant neoplasm of corpus uteri |
|  |  | C61 | Malignant neoplasm of prostate |
|  |  | C64 | Malignant neoplasm of kidney, except renal pelvis |
|  |  | C65 | Malignant neoplasm of renal pelvis |
|  |  | C66 | Malignant neoplasm of ureter |
|  |  | C67 | Malignant neoplasm of bladder |
|  |  | C68 | Malignant neoplasm of other and unspecified urinary organs |
|  |  | C79.0 | Secondary malignant neoplasm of kidney and renal pelvis |
|  |  | C79.1 | Secondary malignant neoplasm of bladder and other and unspecified urinary organ |
|  |  | C17 | Malignant neoplasm of small intestine |
|  |  | C18 | Malignant neoplasm of colon |
|  |  | C19 | Malignant neoplasm of rectosigmoid junction |
|  |  | C20 | Malignant neoplasm of rectum |
|  |  | C21 | Malignant neoplasm of anus and anal canal |
|  |  | C22 | Malignant neoplasm of liver and intrahepatic bile ducts |
|  |  | C23 | Malignant neoplasm of gallbladder |
|  |  | C24 | Malignant neoplasm of other and unspecified parts of biliary tract |
|  |  | C25 | Malignant neoplasm of pancreas |
|  |  | C26 | Malignant neoplasm of other and ill-defined digestive organs |
|  |  | C78.8 | Secondary malignant neoplasm of other and unspecified digestive organs |
|  |  | C48.0 | Retroperitoneum |

When the same patient has both lithiasis and other obstructive process coded, lithiasis is prioritized.
