# Supplementary material for: Factors associated with urinary diversion and fatality of hospitalised acute pyelonephritis patients in France: a national cross-sectional study (FUrTIHF-2)
Source: Epidemiol Infect. 2023 Sep 18;151:e161. doi: 10.1017/S0950268823001504 (PMC10600899; doi:10.1017/S0950268823001504)
Supplement: Grammatico-Guillon et al. supplementary material 2 — Grammatico-Guillon et al. supplementary material [file S0950268823001504sup002.docx]

**Supplementary material B**

**Table. Results of the hospital discharge database coding accuracy estimation** – *Programme de Médicalisation des Systèmes d’Information* PMSI.

| **Clinical gold standard** | **PMSI encoding** | **Sensitivity** [95%CI] | | **Specificity** [95%CI] | **PPV** [95%CI] | **PNV** [95%CI] | **LR+** [95%CI] | **LR-** [95%CI] |
| --- | --- | --- | --- | --- | --- | --- | --- | --- |
| Acute pyelonephritis (AP) | AP code | - | - | | 90.6%  [84.8;94.8] | - | - | - |
| Obstructive AP | Obstruction code | 38.2%  [21.7;54.3] | 97.4%  [92.6;99.4] | | 81.3%  [54.3;96.0] | 84.3%  [77.0;90.0] | 14.78 [4.47;48.88] | 0.63  [0.49;0.83] |
|  | Urinary diversion procedure code | 85.3%  [68.9;95.0] | 99.1%  [95.3;99.9] | | 96.7%  [82.8;99.9] | 95.8%  [90.5;98.6] | 98.94  [13.99;699.97] | 0.15  [0.07;0.33] |
|  | Obstruction code OR urinary diversion procedure code | 97%  [84.7;99.9] | 96.6%  [91.4;99.0] | | 89.2%  [74.6;97.0] | 99.1%  [95.2;99.9] | 28.15  [10.73;73.86] | 0.03  [0.01;0.21] |
| Urinary diversion | Urinary diversion procedure code | 90.9%  [75.7;98.0] | 100%  [96.9;100] | | 100%  [88.4;100] | 97.5% [92.9;99.5] | - | 0.09  [0.03;0.27] |
| *95%CI : 95% confidence interval ; PPV : predictive positive value ; PNV : predictive negative value ; LR+ : positive likelihood ratio ; LR- : negative likelihood ratio* | | | | | | | | |
