# Supplementary material for: Factors associated with urinary diversion and fatality of hospitalised acute pyelonephritis patients in France: a national cross-sectional study (FUrTIHF-2)
Source: Epidemiol Infect. 2023 Sep 18;151:e161. doi: 10.1017/S0950268823001504 (PMC10600899; doi:10.1017/S0950268823001504)
Supplement: Grammatico-Guillon et al. supplementary material 3 — Grammatico-Guillon et al. supplementary material [file S0950268823001504sup003.docx]

**Supplementary material C**

**Case definition of sepsis via hospital discharge databases**

(*Programme de Médicalisation des Systèmes d’Information* PMSI)

1. **Sepsis – *PMSI* case definition**

| **At least one ICD-10 code among:** | | |
| --- | --- | --- |
|  | A40 | Streptococcal sepsis |
|  | A41 | Other sepsis |
|  | A427 | Actinomycotic sepsis |
|  | B377 | Candidal sepsis |
|  | R572 | Septic shock |
|  | R651 | Systemic Inflammatory Response Syndrome of infectious origin with organ failure |

1. **Severe sepsis – *PMSI* case definition**

| **At least one ICD-10 code among:** | | |
| --- | --- | --- |
|  | R572 | Septic shock |
|  | R651 | Systemic Inflammatory Response Syndrome of infectious origin with organ failure |
